# TRANS-FUSIMO -- clinical translation of patient specific planning and conduction of FUS treatment in moving organs

**DOI:** 10.1186/2050-5736-3-S1-O85

**Published:** 2015-06-30

**Authors:** Tobias Preusser, Mario Bezzi, Jürgen Jenne, Thomas Langø, Yoav Levy, Michael Mueller, Giora Sat, Christine Tanner, Stephan Zangos, Matthias Guenther, Andreas Melzer

**Affiliations:** 1 Fraunhofer MEVIS & Jacobs University Bremen, Bremen, Germany; 2 Universita Degli Studi Di Roma La Sapienza, Rome, Italy; 3 Mediri GmbH, Heidelberg, Germany; 4 Stiftelsen Sintef, Trondheim, Norway; 5 InSightec Ltd, Tirat Carmel, Israel; 6 IBSmm Engineering spol. s r. o, Brno, Czech Republic; 7 GE Medical Systems Israel Ltd., Tirat Carmel, Israel; 8 ETH Zurich, Computer Vision Laboratory, Zurich, Switzerland; 9 Johann Wolfgang Goethe-Universität, Frankfurt, Germany; 10 Fraunhofer MEVIS, Bremen, Germany; 11 University of Dundee, Dundee, United Kingdom

## Background/introduction

The movement of the target challenges the application of high intensive focused ultrasound (HiFU/MRgFUS) for the treatment of malignancies in moving abdominal organs such as liver and kidney. Moreover, the anatomical location of the lesion is often behind the rib cage. The physiology of the organs, the dynamic and complex blood perfusion impairs the energy disposition in the tissue due to the heat transfer within the organ. To explore the full potential of extracorporeal FUS to safely and precisely destroy tissue in the depth of a moving organ requires sophisticated software and advanced hardware. In the EU project FUSIMO (http://www.fusimo.eu) a software demonstrator for the patient specific planning of FUS in the liver has been developed in order to empower the physician to perform safe, effective and efficient ablation of tumours and to facilitate prediction of the outcome. The new EU project TRANS-FUSIMO (http://www.trans-fusimo.eu) aims at the translation of this software demonstrator into a fully integrated system for the FUS treatment of the liver.

## Methods

The FUSIMO software demonstrator based on MeVisLab incorporates a set of dynamic organ models for the physical and biophysical processes involved in MR guided FUS treatment: (i) an organ motion model simulates the patient specific deformation of the relevant anatomical structures during breathing; (ii) a patient specific tissue model represents the ultrasound propagation, the energy distribution as well as the tissue heating and cooling; (iii) an organ/tumour model captures the patient specific tissue’s response to the therapy. These model components are integrated into a software demonstrator, which orchestrates the interplay between the models and feeds them with model parameters that are extracted from patient specific MR and/or US imaging data. The system and the model components are being validated in phantom and *ex vivo* experiments and in Thiel soft embalmed human cadavers. In TRANS-FUSIMO the safety, efficacy and efficiency of the software assistant will be evaluated in an *in vivo* animal study. Moreover, a two-arm study (neoadjuvant TRANS-FUSIMO MRgFUS + resection, TRANS-FUSIMO MRgFUS only) for human patients with metastases or HCC will show the feasibility of the TRANS-FUSIMO system for the clinical setting.

## Results and conclusions

The FUSIMO/TRANS-FUSIMO software demonstrator comprises specific models for the simulation of FUS application in moving organs based on imaging data derived from volunteers. It supports the assessment of the feasibility of the intervention, predicting and optimizing the outcome, detecting potential risks and avoiding them, as well as monitoring the progress and tracking deviations from the planned procedure. Our *ex vivo* experiments show that the FUSIMO system is capable of compensating organ motion through real-time motion detection, motion modelling and real-time beam steering.

In TRANS-FUSIMO a fully integrated system will be developed for which *in vivo* animal studies and first patient study shall show that MRgFUS in moving organs can be performed safely, efficaciously and effectively.

